# Characteristics of groundwater microbial communities and the correlation with the environmental factors in a decommissioned acid *in-situ* uranium mine

**DOI:** 10.3389/fmicb.2022.1078393

**Published:** 2023-02-22

**Authors:** Fangfang Zhu, Bei Zhao, Wenwen Min, Jiang Li

**Affiliations:** ^1^State Key Laboratory of Nuclear Resources and Environment, East China University of Technology, Nanchang, Jiangxi, China; ^2^School of Water Resources and Environmental Engineering, East China University of Technology, Nanchang, Jiangxi, China; ^3^School of Water Resources and Environment, China University of Geosciences (Beijing), Beijing, China; ^4^School of Information Science and Engineering, Yunnan University, Kunming, Yunnan, China; ^5^School of Chemistry, Biology and Materials Science, East China University of Technology, Nanchang, Jiangxi, China

**Keywords:** decommissioned uranium mine, acid *in-situ* leaching, groundwater, microbial community, environmental factors

## Abstract

Microorganisms play an important role in the bioremediation process for the decommissioned acid *in-situ* leaching uranium mine. It is crucial to understand the original microbial community characteristics before the *in-situ* bioremediation. However, there are limited studies on the groundwater microbial characteristics in the decommissioned acid *in-situ* uranium mine. To this end, we collected groundwater samples, including the groundwater that originally residual in the borehole (RW) and the aquifer water (AW), from a decommissioned acid *in-situ* uranium mine in the southern margin of Ili Basin in Xinjiang, China. The occurrence characteristics of the groundwater microbial communities and their correlation with environmental factors were systematically studied based on the high throughput 16S rRNA gene sequencing data and geochemical data. Results found that the AW samples had higher alpha- and beta- diversity than the RW samples. The relative abundance of *Sporosarcina*, *Sulfobacillus*, *Pedobacter* and *Pseudomonas* were significantly different in the AW and RW samples, which had significant correlation with pH, metals, and sulfate, etc. A series of reducing microorganisms were discovered, such as sulfate reduction (e.g., *Desulfosporosinus*) and metal reduction (e.g., *Arthrobacter*, *Bacillus*, *Clostridium*, *Pseudomonas*, and *Rhodanobacter*), which have the potential to attenuate sulfate and uranium in groundwater. In addition, we found that pH and redox potential (Eh) were the dominant environmental factors affecting the microbial composition. This study extends our knowledge of microbial community structure changes in the decommissioned acid *in-situ* uranium mine and has positive implications for assessing the potential of natural attenuation and bioremediation strategies.

## 1. Introduction

Uranium *in-situ* recovery (ISR) or *in-situ* leaching (ISL) refers to a uranium mining method that directly leaches target components from uranium-bearing ore body without changing the spatial location of uranium ore under natural conditions. It was widely applied in the mining of sandstone-type uranium ore (Taylor et al., 2004). ISL typically using sulfuric acid as a leaching agent to extract uranium from host sandstones (Mudd, 2001; Taylor et al., 2004), thereby increasing the concentration of dissolved solids in groundwater. With the decommissioning of uranium mines, the existed sulfuric acid and various heavy metals would pose a serious threat to the groundwater ecosystem in the mining areas (Saunders et al., 2016). Heavy metals generally had obvious biotoxicity, and adverse effects on the environment and human beings through bioaccumulation and biomagnification in the food chain (Naikoo et al., 2021). And they were difficult to biodegrade, thus causing persistence features (Ali et al., 2019). Consequently, the migration of such toxic aqueous metal cations would pose a potential threat to human health and other ecosystems. Therefore, the remediation of groundwater in decommissioned acid in-situ uranium mine has become an urgent environmental problem (Mudd, 2001; Riedel and Kübeck, 2018).

Compared with physical and chemical remediation, bioremediation has the advantages of economy and no secondary pollution, and is widely used in the treatment of environmental pollution (Adams et al., 2015). Microorganisms are key organisms in many elemental cycles on Earth, such as carbon, nitrogen, and sulfur cycles, and strongly influence the transport of various heavy metals (Wegner et al., 2019; Bell et al., 2020; Rogiers et al., 2022). Understanding the interaction between microorganisms and pollutants will provide insights into the mechanism of environmental factors affecting microbial degradation and corresponding biogeochemical processes (Cho et al., 2012; Varjani et al., 2017). Therefore, studying the composition, the distribution and remediation potential of indigenous microbial community in the groundwater of decommissioned acid *in-situ* uranium mine can provide theoretical support for the bioremediation of uranium related pollution.

The groundwater microorganisms often present different distribution characteristics in different stages of uranium mining, including pre-mining (Jroundi et al., 2020), acid leaching mining stage (Coral et al., 2018), and post in-situ uranium leaching stage (Cho et al., 2012; Green et al., 2012). For instance, some studies have shown that microbial communities in uranium mine groundwater using acid-ISL are similar with those in acid mine drainage (AMD), mainly including *Sulfobacillus*, *Leptospirillum. Acidithiobacillus* (Méndez-García et al., 2015; Coral et al., 2018). With the decommissioning of uranium mines, various reducing microorganisms gradually increased (Cho et al., 2012; Green et al., 2012), including denitrifying bacteria such as *Rhodanobacter* (Tao et al., 2022), and the microorganisms that possibly involved in metal reduction, such as *Shewanella*, *Pseudomonas*, *Stenotrophomonas* (Wall and Krumholz, 2006; You et al., 2021). In addition, these microorganisms may also be involved in other respiratory metabolism, such as nitrate reducers, iron reducers or thiosulfate reducers. For example, *Pseudomonas* was reported have the ability of nitrate reduction (Huang et al., 2020).

At present, there are many studies on the correlation between microbial community structure and environmental factors in uranium mining area (Cho et al., 2012; Green et al., 2012; Coral et al., 2018), including active acid leaching uranium aquifer(Coral et al., 2018), uranium-contaminated tailing ponds (Dhal, 2018), former leaching heap area (~26 years;Sitte et al., 2015), and decommissioned uranium mine aquifers characterized by NO_3_^−^ and uranium contamination (Cho et al., 2012; Green et al., 2012). In addition, many studies have also reported the dynamic changes of microbial communities during bioremediation (Zhang et al., 2017; Tang et al., 2021). However, there are limited studies about the characteristics of microbial communities in groundwater of decommissioned acid *in-situ* uranium mine. Therefore, we selected a sandstone type uranium deposit using acid ISL in Northwest China as the research area, which has been decommissioned for nearly 20 years. The groundwater environment in the mining area is characterized by low pH and high concentration of sulfate and heavy metals. In addition, we selected two types of groundwater samples, including the residual water (RW) and the aquifer water (AW). The RW sample is the groundwater originally left in the borehole, while AW refers to the aquifer water after pumping twice the volume of water in the pipe. Due to poor connectivity with aquifer, the mobility of the groundwater remaining in the borehole is lower than that in the aquifer. And RW is in long-term contact with the air, and may even be recharged by a small amount of rain (Coulson et al., 2022). These often lead to obvious difference between the water quality of the residual water (RW) and the aquifer water (AW). However, few studies have compared the water quality and microbial communities in these two types of groundwater, which is helpful to understand the natural attenuation process and microbial communities’ role in the hydrogeochemical element cycle.

Our main contributions of this paper are summarized as follows: (1) Study the composition and diversity of microbial communities in two types of groundwater of decommissioned acid in-situ uranium mine in the southern margin of Ili Basin in Xinjiang, China; (2) Identify some differential microbes between the AW and RW groundwater samples; (3) Explore the relationship between microbial communities and environmental factors and identify some dominant environmental factors.

## 2. Materials and methods

### 2.1. Site description

The study area is located on a decommissioned uranium mining area in the southern margin of Ili Basin in Xinjiang, China. The uranium deposit is the first large sandstone type uranium deposit in China that has successfully applied acid in-situ leaching mining technology. After nearly 30 years of acid in-situ leaching industrial test and production. Many old mining areas of the uranium deposit have been decommissioned one after another. The boreholes in the study area have been decommissioned for nearly two decades after mining for about 10 years.

### 2.2. Groundwater sampling and in-situ measurements

**Figure 1:**
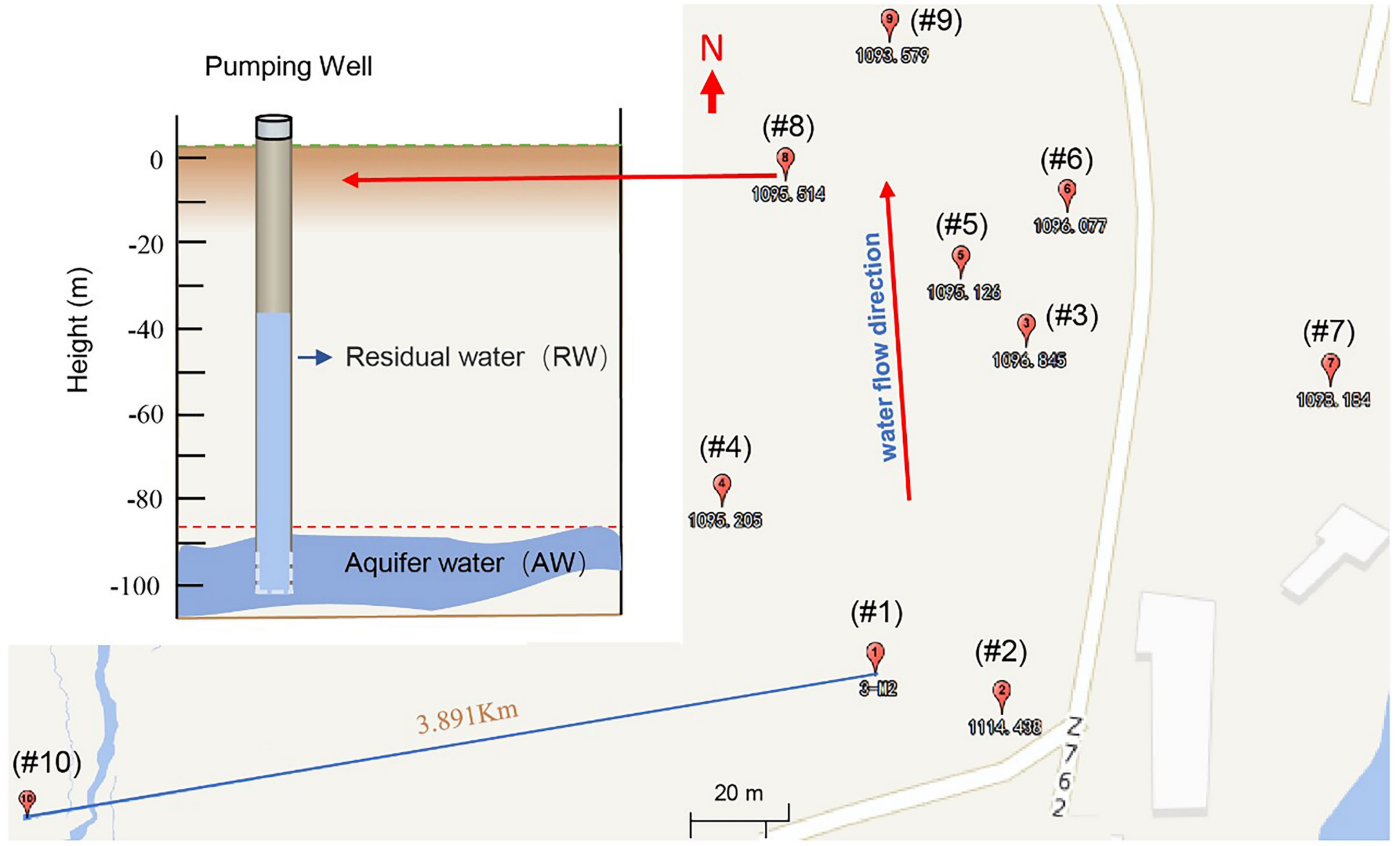
Groundwater sampling locations of a uranium mining area in the southern margin of the Ili Basin, Xinjiang in China. Sampling well number from #1 to #9 (the subscript number is the standard height in meters, where each well has two sampling points, namely the Residual Water (RW) and the Aquifer water (AW) (See the upper left). The RW sample is the groundwater originally left in the hole, while AW refers to the aquifer water after pumping twice the volume of water in the pipe. In addition, we also took a sample for the background value (#10), which represented the natural state of the study area before mining, located on the periphery of the mining area.

In October 2021, a total of 19 groundwater samples were collected from 10 boreholes, including 18 samples from 9 boreholes in the contaminated area, as well as 1 sample in background site (BW; Figure 1). Firstly, we sampled the residual water in the tube well, then pumped water for 2 to 3 h, extracted groundwater three times the volume of residual water, and waited for the aquifer water to enter the tube well before sampling. The temperature (T), pH, redox potential (Eh), electrical conductivity (EC) and dissolved oxygen (DO) of groundwater samples were measured onsite within 10 min after sampling using a portable multi-probe instrument (Hach, SL1000). Groundwater for cations analysis was acidified to pH < 2.0 using HNO_3_. Samples for geochemical measurements were filtered through a pre-sterilized 0.22 μm filter and kept at below 4°C during the field-work. For the molecular biological analysis, groundwater (5 ~ 10 l) was filtered through a pre-sterilized 0.22 μm filter to collect biomass and stored at −80°C until use.

### 2.3. Laboratory geochemical analysis

The groundwater samples were all sent to Beijing Research Institute of Uranium Geology for geochemical analysis. Anions, including F^−^, Cl^−^, NO_3_^−^, SO_4_^−^, NO_2_^−^, were measured by ion chromatography (883 Basic IC plus analyzer, Switzerland). Molybdenum blue photometric method (UV-POWER, China) was applied to measure PO_4_^3−^. The Cations, including Ca^2+^, Mg^2+^, K^+^, Na^+^, Al^3+^, As, Ba^2+^, Cd^2+^, Cr, Cu^2+^, Fe, Mn, Mo, Pb, U, Zn^2+^, were measured by Inductively Coupled Plasma Mass Spectrometry (ICP-MS, ELEMENT XR, Thermo Scientific, America) and Inductively Coupled Plasma Optical Emission Spectrometer (ICP-OES, 5300DV, America).

### 2.4. DNA extraction and PCR amplification

Microbial community genomic DNA was extracted from the collected groundwater samples using the E.Z.N.A.® soil DNA Kit (Omega Bio-tek, Norcross, GA, U.S.) according to manufacturer’s instructions. The DNA extract was checked on 1% agarose gel, and DNA concentration and purity were determined with NanoDrop 2000 UV–vis spectrophotometer (Thermo Scientific, Wilmington, USA). The hypervariable region V3-V4 of the bacterial 16S rRNA gene were amplified with primer pairs 338F (5′-ACTCCTACGGGAGGCAGCAG-3′) and 806R (5′-GGACTACHVGGGTWTCTAAT-3′; Mori et al., 2014) by an ABI GeneAmp® 9,700 PCR thermocycler (ABI, CA, USA). The PCR amplification of 16S rRNA gene was performed as follows: initial denaturation at 95C for 3 min, followed by 27 cycles of denaturing at 95°C for 30 s, annealing at 55°C for 30 s and extension at 72°C for 45 s, and single extension at 72°C for 10 min, and end at 10°C. The PCR mixtures contain 5 × *TransStart* FastPfu buffer 4 μl, 2.5 mM dNTPs 2 μL, 5 μM forward primer 0.8 μL, 5 μM reverse primer 0.8 μL, *TransStart* FastPfu DNA Polymerase 0.4 μL, template DNA 10 ng, and finally added ddH_2_O up to 20 μL. PCR reactions were performed in triplicate. The PCR product was extracted from 2% agarose gel and purified using the AxyPrep DNA Gel Extraction Kit (Axygen Biosciences, Union City, CA, USA) according to manufacturer’s instructions and quantified using Quantus™ Fluorometer (Promega, USA).

### 2.5. Illumina MiSeq sequencing and processing of sequence data

Purified amplicons were pooled in equimolar and paired-end sequenced on an Illumina MiSeq PE300 platform (Illumina, San Diego, USA) according to the standard protocols by Majorbio Bio-Pharm Technology Co. Ltd. (Shanghai, China). The raw 16S rRNA gene sequencing reads were demultiplexed, quality-filtered by fastp version 0.20.0 (Chen et al., 2018) and merged by FLASH version 1.2.7 (Magoč and Salzberg, 2011) with the following criteria: (i) the 300 bp reads were truncated at any site receiving an average quality score of less than 20, and the truncated reads shorter than 50 bp or reads containing ambiguous characters were discarded; (ii) only overlapping sequences longer than 10 bp were assembled according to their overlapped sequence. The maximum mismatch ratio of overlap region is 0.2. Reads that could not be assembled were discarded; (iii) Samples were distinguished according to the barcode and primers. Operational taxonomic units (OTUs) with 97% similarity cutoff (Stackebrandt et al., 1994; Edgar, 2013) were clustered using UPARSE version 7.1 (Edgar, 2013), and chimeric sequences were identified and removed. The taxonomy of each OTU representative sequence was analyzed by RDP Classifier version 2.2 (Wang et al., 2007) against the 16S rRNA database using confidence threshold of 0.7.

### 2.6. Statistical analysis

#### 2.6.1. Alpha diversity

Alpha diversity is used to measure the diversity within a sample. In our study, we apply multiple alpha diversity indicators of microbial communities, including the observed richness (Sobs), Ace (an index used to estimate the number of OTUs in a community), Chao, Shannon, and Simpson index (Finotello et al., 2018).

#### 2.6.2. Beta diversity and PCoA

Beta diversity is a term used to express the differences between samples, which is very often used in microbiome studies to help researchers see whether there are major differences between two groups (Finotello et al., 2018). Principal coordinates analysis (PCoA; Gower, 2014) is a non-constrained data dimensionality reduction analysis method, which can be used to study the similarity or difference of samples. The main difference between PCoA and Principal component analysis (PCA) is that PCA uses the taxa (including OTU) abundance table to map directly based on Euclidean distance, while PCoA derives from Bray-Curtis distances among samples, both of which are potential principal components that affect the differences in sample community composition through dimensionality reduction. The algorithm for the Bray-Curtis distance matrix is:


$$D_{Bray-Curtis}=1-2\frac{\min\left(S_{A,i}+S_{B,i}\right)}{\sum S_{A,i}+\sum S_{B,i}}$$

where $S_{A,i}$ represents the sequence number counted of i taxon in sample A and $S_{B,i}$ represents that of in sample B .

#### 2.6.3. Distance-based redundancy analysis

db-RDA is currently the most widely used environmental factor analysis method to reveal the relationship between environmental variables and microbial communities (Legendre and Anderson, 1999).

#### 2.6.4. Spearman correlation analysis

Spearman correlation analysis is also called Spearman Rank Correlation Analysis, which is a nonparametric statistical method. In this study, it was used to evaluate the relationship between environmental factors and taxa diversity. The Spearman correlation coefficient r has a value between −1 and + 1 (Bonett and Wright, 2000).

#### 2.6.5. Partial Least Squares Discriminant Analysis

PLS-DA is a Statistical technique which can effectively discriminate between group observations and find the influencing variables that lead to the difference between groups by properly rotating the principal components. PLS-DA adopts the classical partial least squares regression model, and its response variable is a group of categorical information that reflects the category relationship between statistical units, which is a supervised discriminant analysis method (Lee et al., 2018).

#### 2.6.6. LEfSe phylogenetic dendrogram

A non-parametric factorial Kruskal–Wallis (KW) sum-rank test was used to detect features with significant differences in abundance, and then Linear Discriminant Analysis (LDA) was employed to estimate the magnitude of the effect of each taxon abundance on the differential effect.

#### 2.6.7. Computing platform

The data analysis was performed using the online platform of Majorbio Cloud Platform^1^ (Ren et al., 2022) and R software packages.^2^

## 3. Results

In this study, we collected 19 groundwater samples and the corresponding microbial count data based on the high throughput 16S rRNA gene sequencing method were presented in Supplementary Tables S1–S6.

### 3.1. Environmental factors characterization of groundwater

**Figure 2:**
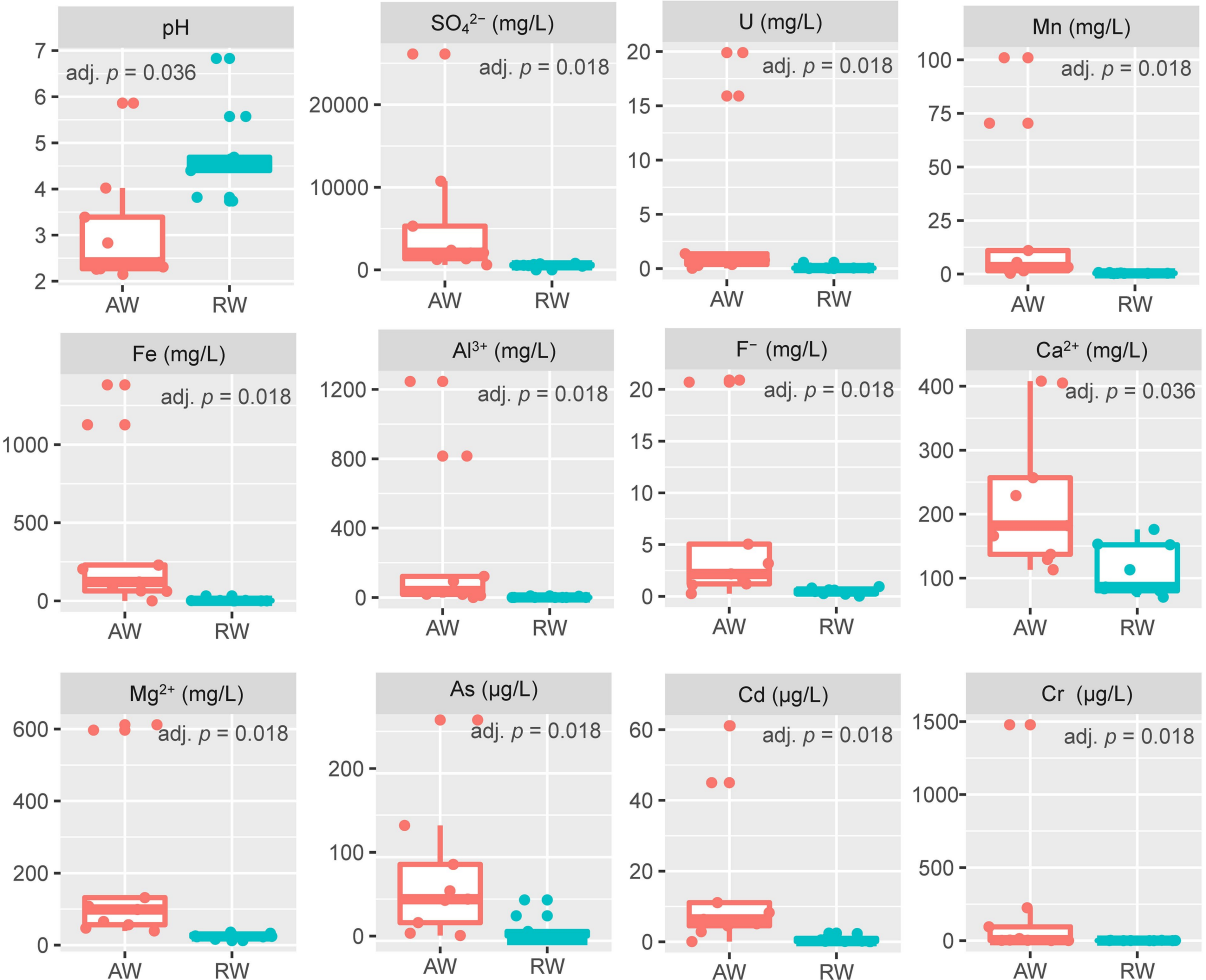
Boxplot of environmental factors between the two sample types (AW and RW). For the values of each environmental factor between the AW and RW samples, we used the non-parametric Wilcoxon signed-rank test to evaluate their differences and the corresponding Benjamini and Hochberg (BH) adjusted *p*-value (adj. *p*) was calculated.

For the collected groundwater samples in this study, a total of 27 environmental factors were measured by *in situ* testing and laboratory analysis (Supplementary Table S7). Some environmental factors with significant differences between the AW and RW samples are shown in Figure 2. The pH values of AW samples were between 2.15 and 5.86 with average 2.94. The pH values of RW samples were between 3.82 and 6.83 with average 4.75. Statistically the average pH of the AW samples tends to be lower than the average pH of the RW samples (adj. *p* = 0.036). The Eh values of AW samples were generally higher than that of the corresponding RW samples, except for AW_3 and AW_5. Among the metal(loid)s, U, Mn, Fe, Al^3+^, Cr, As, Cd, Ca^2+^ and Mg^2+^ were significantly (with *p* < 0.05) higher in the AW than in RW (Figure 2). The average concentrations of U, Fe, Mn, Cr, Cd, As in AW samples were 4.456, 364, 22, 0.2, 0.016, 0.07 mg/L, respectively, and approximately 45, 80, 51, 278, 21, 8 times higher than that in RW samples, respectively. The main reason was that the heavy metal elements in the ore-bearing layers were continuously dissolved into the groundwater caused by the extremely acidic environment, resulting in a high degree of water pollution in the AW samples. Moreover, the average concentrations of SO_4_^2−^, F^−^ in AW samples were 5,763 and 6.2 mg/L, respectively, which were 11 and 13 times higher than that in the RW samples (Figure 2).

The concentrations of most metal elements and anions in AW_3 was much lower than other AW samples, but close to the RW samples and BW sample. The concentration of U in AW_2 was the highest (19.9 mg/L), followed by AW_1 (15.9 mg/L), while that of other AW samples were 0.026 ~ 1.37 mg/L. The concentration range of U in RW samples were 0.006 ~ 0.567 mg/L. SO_4_^2−^ occurred the highest concentration in AW_1 (26,132 mg/L), followed by AW_2 (10,739 mg/L), while that other AW samples occurred with a range of 613 ~ 5,299 mg/L. The concentration range of SO_4_^2−^ in RW samples were 443 ~ 790 mg/L. The concentrations range of Fe in most AW samples were 61.1 ~ 1,382 mg/L (AW_3, 0.115 mg/L), while in RW samples were 0.015 ~ 32.1 mg/L. The concentration of Mn in AW_1 was the highest (101 mg/L), followed by AW_2 (70.4 mg/L), with a range of 0.364 ~ 11 mg/L in other AW samples and 0.207 ~ 0.74 mg/L in the RW samples. The pH and Eh values in BW sample was 6.92 and − 53.9, respectively, which represented the natural state of the study area before mining. Similarly, the concentrations of heavy metals and anions in this sampling site can also be used as natural background values to compare with those in contaminated site. In addition, the Eh values of two boreholes in the adjacent active acid leaching area were detected, which were 437.6 mv and 442.2 mv, respectively.

### 3.2. Microbial diversity and community structure of groundwater

#### 3.2.1. Alpha diversity of microbial communities

**Figure 3:**
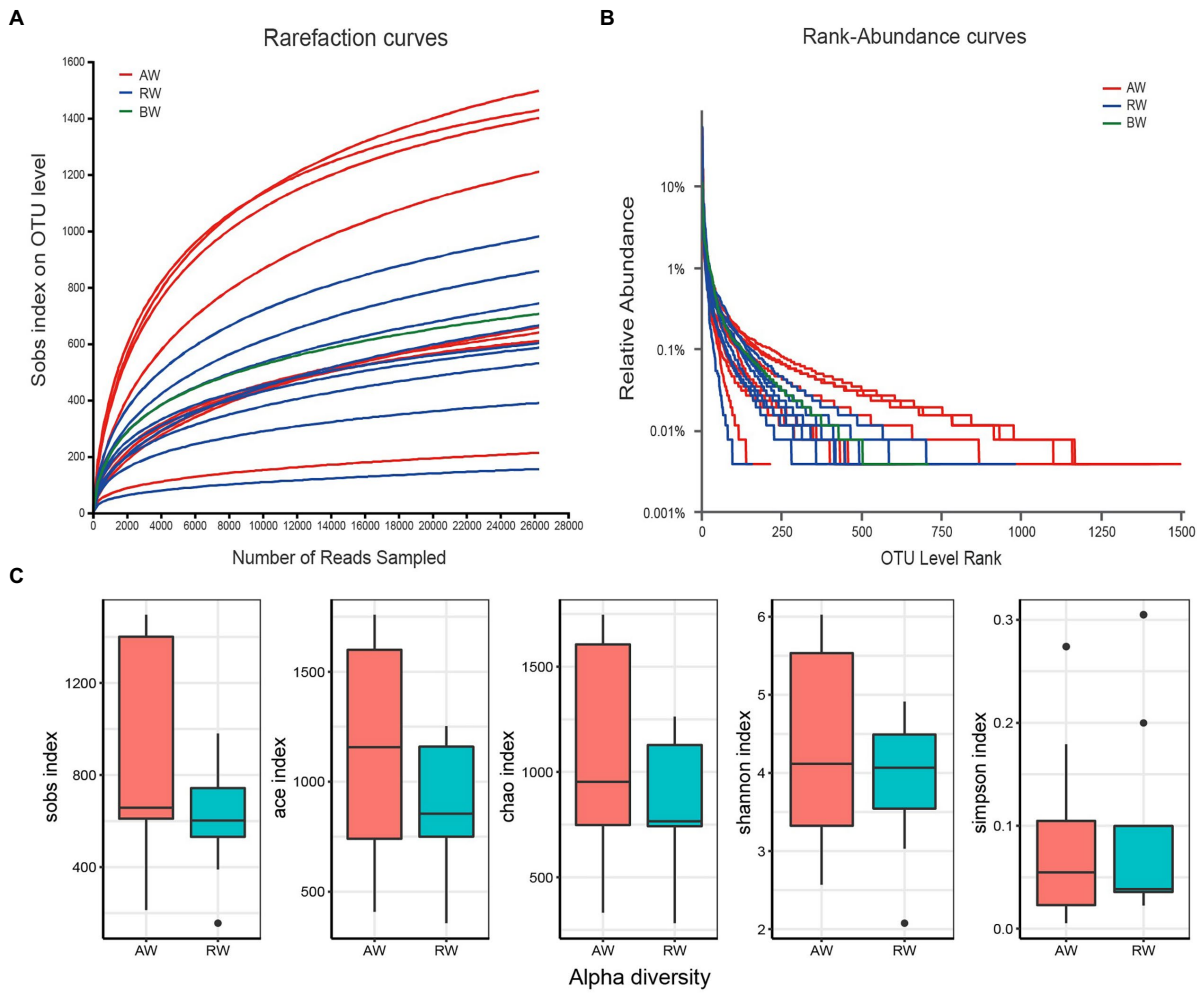
**(A)** Rarefaction curves at different sequencing depths. The horizontal axis represents the amount of randomly sampled sequencing data and the vertical axis represents the number of observed taxa (i.e., Sobs). “BW” represents background sample. **(B)** Relative abundance of the ranking OTU sequences in the groundwater samples. **(C)** Distribution boxplot of the alpha diversity index among two groups.

The rarefaction curves showed that there was a sufficient sequencing depth for microbial community analysis (Figure 3A). The relative abundance of OTU showed that the taxa richness of AW samples was generally higher than that of RW samples, with the groundwater sample at the background site in the middle (Figure 3B). Furtherly, the Alpha-diversity indexes of microorganisms in AW and RW samples were calculated (Supplementary Table S8; Figure 3C), including Shannon, Chao, Sobs, Ace and Simpson index. The results showed that the five diversity indexes in AW samples were generally higher than that of RW samples (Figure 3C). Because microbial diversity is an important indicator which can describe the number of different taxa of microbes present and their distribution. To study which environmental factors may affect the microbial diversity in this study area, we computed the spearman correlation coefficient between the microbial diversity indexes and environment factors. And the results showed that the sobs, ace, chao and Shannon index had significantly positive correlations with environmental factors Ba (*p* < 0.05; Supplementary Table S9).

#### 3.2.2. Compositional analysis of groundwater microbial community structures

**Figure 4:**
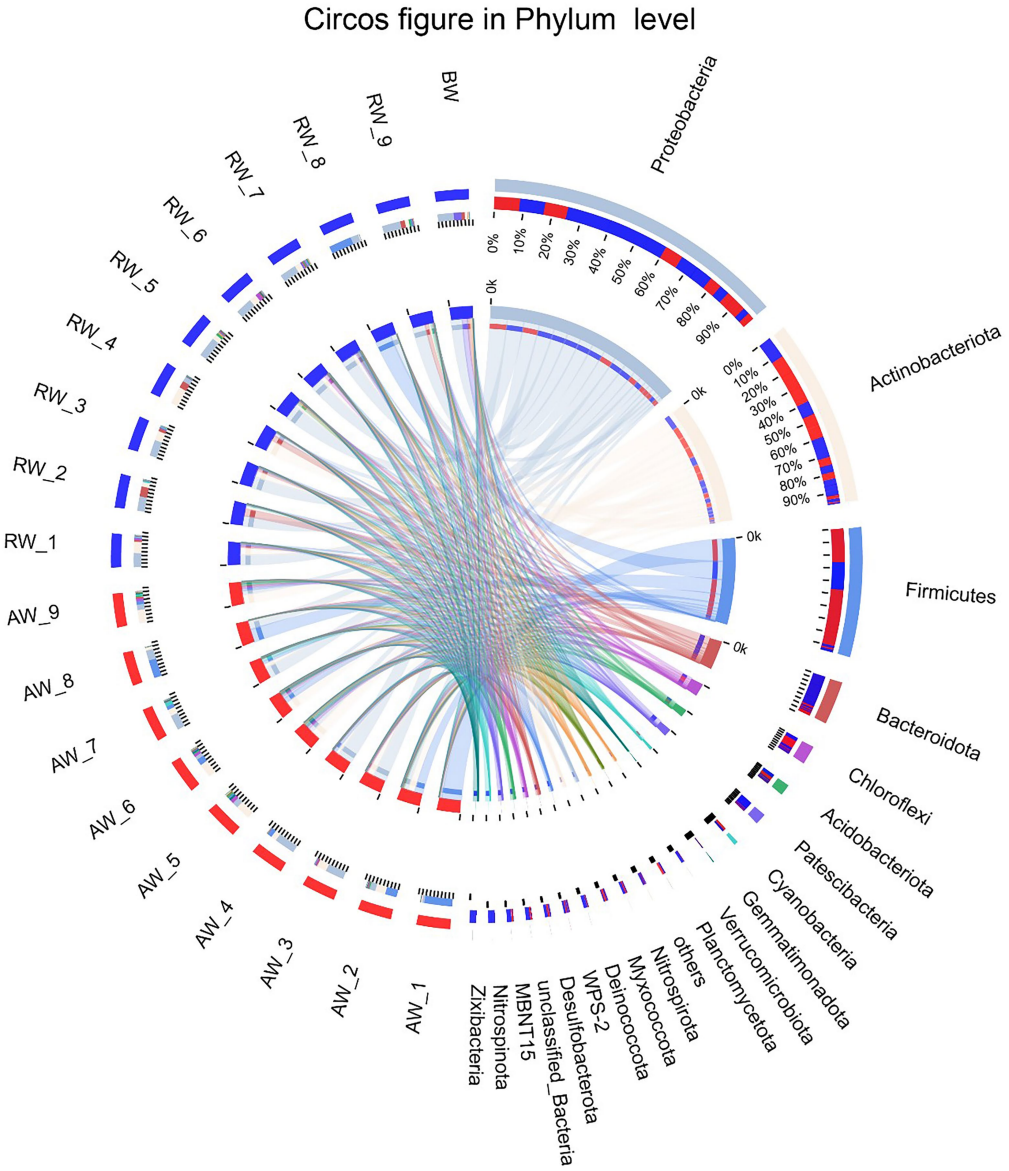
The Circos diagram of sample-taxa relationship in Phylum level. The inner circle represents the composition of taxa in the sample, the color of the inner ribbon represents the taxa, and its length represents the relative abundance of the taxa in the corresponding samples. The outer circle represents the distribution ratio of taxa in different samples at the Phylum level, the outer ribbon represents taxa, the inner ribbon color represents different groups, and the length represents the distribution ratio of the sample in a taxon.

**Figure 5:**
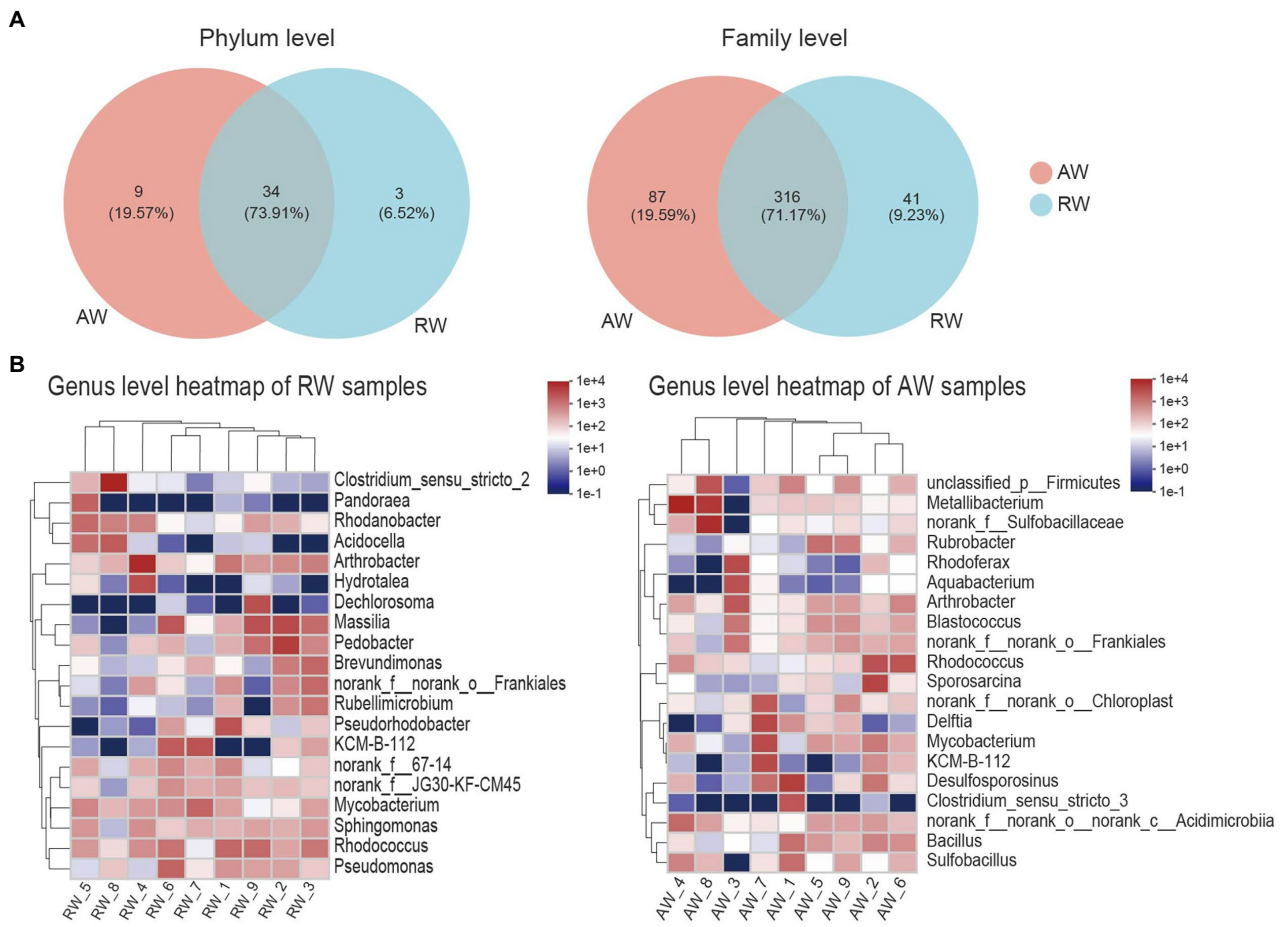
Taxa composition analysis in phylum, family and genus level. **(A)** Venn diagram of the microorganisms in phylum and family level, the different colors represent different groups and the numbers in the overlapping part represent the number of taxa shared by two groups, and the numbers in the non-overlapping part represent the number of taxa unique to the corresponding group. **(B)** Heatmap of the top 20 families of relative abundance at genus level.

To perform microbial community compositional analysis for the collected groundwater samples, the microbial community structures at phylum and family level were analyzed, respectively (Figures 4, 5A). A total of 46 phyla were detected in 18 samples, with 43 phyla in AW samples and 37 phyla in RW samples (Supplementary Figure S1). The most abundant 10 phyla accounted for 97% of the total abundance. And the top five phyla accounted for 90.6%, which were *Proteobacteria* (38%), *Actinobacteriota* (25.2%), *Firmicutes* (18%), *Bacteroidota* (6%), and *Chloroflexi* (3.4%), respectively (Figure 4). A total of 23 phyla were detected in the BW sample, the most abundant 10 phyla accounted for 96.3%, with the top three phyla accounted for 84.5%, which were *Proteobacteria* (50%), *Patescibacteria* (23.9%), and *Bacteroidota* (10.6%), respectively. Both *Proteobacteria* and *Actinobacteriota* were the dominant phyla in the AW and RW samples, while *Firmicutes* and *Bacteroidota* have significant differences between them (Supplementary Figure S2).

At genus level (Figure 5B), *Arthrobacter*, *norank_f__norank_o__Frankiales*, *KCM-B-112*, *Mycobacterium*, *Rhodococcus* had high relative abundances in both RW and AW samples. While *Clostridium_sensu_stricto_2*, *Pandoraea*, *Rhodanobacter*, *Acidocella*, *Hydrotalea*, *Dechlorosoma*, *Massilia*, *Pedobacter*, *Brevundimonas*, *Rubellimicrobium*, *Pseudorhodobacter*, *Pseudomonas* only showed high proportion in RW samples. *Metallibacterium*, *norank_f__Sulfobacillaceae, Sulfobacillus*, *Rubrobacter*, *Rhodoferax*, *Aquabacterium*, *Blastococcus*, *Sporosarcina*, *Delftia*, *Desulfosporosinus*, *Clostridium_sensu_stricto_3*, *Bacillus* only had high relative abundance in AW samples. Similarly, *Sulfobacillus*, *Metallibacterium*, *norank_f__Sulfobacillaceae* and *Clostridium_sensu_stricto_3* was also not detected in AW_3. It has been reported that *Metallibacterium* can increase the pH value in acidic environments by producing ammonium and form a microenvironment to enhance the adaptability to different pH conditions (Bartsch et al., 2017). *Desulfosporosinus* had high relative abundance in the AW_1(23.66%), AW_2(5.3%), AW_7(6.3%), respectively. It belongs to *Firmicutes* and was the dominant sulfate-reducing bacteria in AW samples.

#### 3.2.3. Beta diversity of microbial community structure

**Figure 6:**
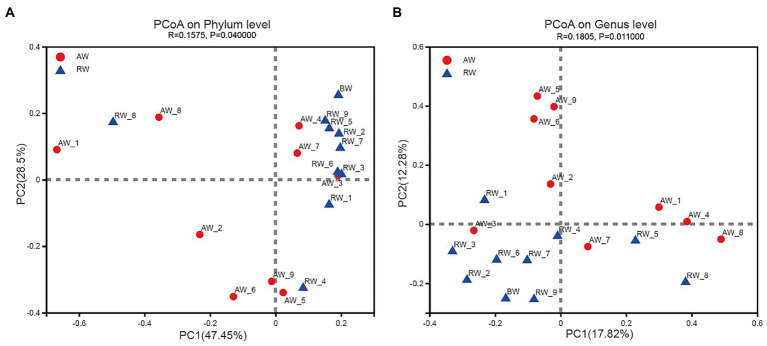
Beta diversity analysis in Phylum and Genes level. Principal Coordinates Analysis (PCoA) derived from Bray-Curtis distances among of AW, RW, and BW samples (*p* < 0.05 by ANOSIM (Analysis of similarities)) and different colored dots represent samples from different groups.

The PCoA method were applied to analyze the similarity of microbial communities at phylum and genus level between AW and RW (Figure 6). The degree of dispersion among the AW samples was significantly higher than that of the RW samples at phylum level (Wilcoxon rank sum test *p*-value = 0.019) and genus level (*p*-value = 0.039). Most of the RW samples were distributed in the first quadrant, while AW samples were evenly distributed in each quadrant (Figure 6A). At phylum level, the microbial composition of RW_8 sample was quite different from other RW samples, which may be due to the large proportion of *Firmicutes* (71.8%) in RW_8. The Bray-Curtis between AW_3 and RW_3 samples were very close, indicating that their microbial compositions were similar. The microbial composition of AW_3, AW_4 and AW_7 samples were similar with that of RW samples at phylum level (Figure 6A). Similarly, both AW_3 and AW_7 samples were relatively close to the RW samples at genus level (Figure 6B). The groundwater samples could be divided into two subgroups. Two main factors made the biggest contribution for microbial composition differences between AW and RW at phylum level, which accounted for 47.45 and 28.5%, respectively. In addition, the correlation coefficient (r) showed that there was significant difference about the microbial communities between the AW and RW samples (r = 0.1575, *p* < 0.05), which was computed based on the Bray-Curtis distance algorithm (Figure 6A).

At genus level, the groundwater samples could also be divided into two categories, with two main factors accounting for 17.82 and 12.28%, respectively. The microbial composition of the two groups of samples were also significantly different at genus level (r = 0.1805, p < 0.05; Figure 6B). The above statistical results showed that the microbial composition of RW samples tended to be more similar, while there was no such trend among the AW samples, which were more different. This was mainly due to the large differences for environmental factors in AW samples, especially the environmental factors at the three points AW_1, AW_2, and AW_3. The first two points were in an extremely acidic environment, containing high concentrations of heavy metals and sulfate ions. While the environmental factors of AW_3 were closer to the BW sample.

### 3.3. Difference analysis of microbes between RW and AW samples

**Figure 7:**
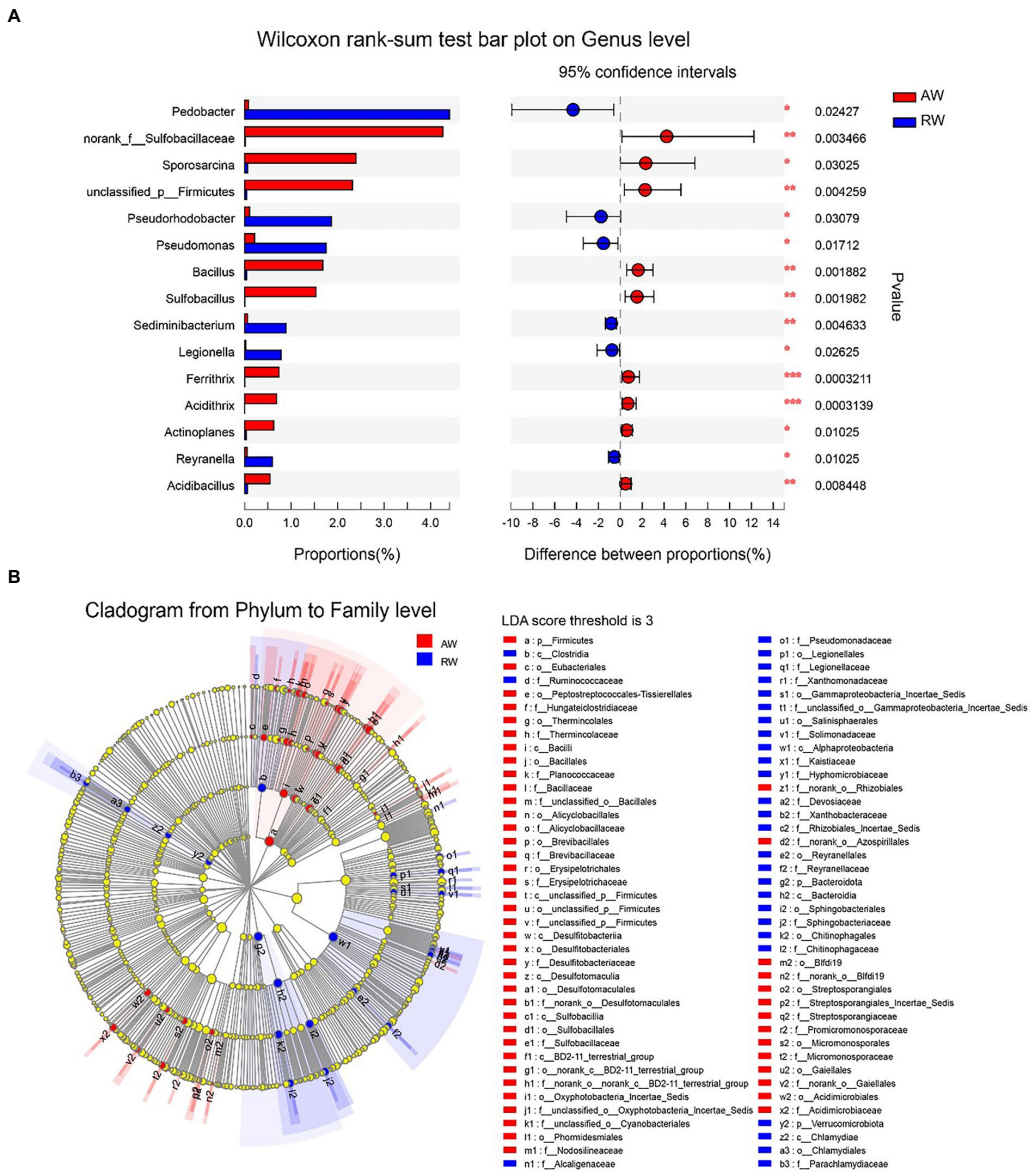
Difference analysis of microbes between RW and AW samples. **(A)** Wilcoxon rank-sum test bar plot on genus level. Only the differentially abundant genus is shown. The *y*-axis represents the taxa name, the *x*-axis represents the average relative abundance of taxa in different groups, and the far right is the corresponding *p*-value, where *0.01 ≤ *p* ≤ 0.05, **0.001 ≤ *p* ≤ 0.01, ***p ≤ 0.001. **(B)** LEfSe Phylogenetic dendrogram of biomarker bacteria between RW and AW samples. We first used the non-parametric factorial Kruskal–Wallis (KW) sum-rank test to detect features with significant differences in abundance, and then employed Linear Discriminant Analysis (LDA) method to estimate the magnitude of the effect of each taxa abundance on the differential effect.

We employed a statistical method to identify the differential microbes between the AW and RW samples (Figure 7). Firstly, the Wilcoxon rank-sum test statistical method was used to calculate the difference level of the relative abundance of bacteria at genus level and 15 genera were discovered with significance difference level of *p* < 0.05 (Figure 7A). *Pedobacter*, *Pseudorhodobacter*, *Pseudomonas*, *Sediminibacterium*, *Legionella*, and *Reyranella* had higher relative abundance levels in RW samples than that in AW samples, while nine genera (including *norank_f_Sulfobacillaceae*, *Sporosarcina*, *unclassified_p_Firmicutes*, *Bacillus*, *Sulfobacillus*, *Ferrithrix*, *Acidithrix*, *Actinoplanes*, and *Acidibacillus*) had higher relative abundance levels in AW samples than that in RW samples. The results of the differential abundance analysis on family level are shown in Supplementary Figure S3. In addition, LEfSe Phylogenetic dendrogram was used to show the microbes biomarkers between RW and AW samples (Figure 7B). We found that more microbes biomarkers were enriched in AW samples than in RW samples.

Furthermore, a total of 11 microorganisms related to sulfur /sulfate reduction were detected in groundwater samples at genus level (Barton and Fauque, 2009), including *Desulfosporosinus*, *Desulfurispora*, *Desulfitobacterium*, *Desulfatirhabdium*, *Desulfobacca*, *Desulfovibrio*, *Desulfurivibrio*, *Desulfurispora*, *Desulfuromusa*, *Desulfatiferula*, and *Desulfotomaculum*. Most of the sulfur- or sulfate-reducing bacteria (SRB) genera identified in the present study showed low abundances in the AW samples and RW samples (Supplementary Figure S4). For example, *Desulfosporosinus,* a major SRB in RW and AW samples, only had relatively high abundance in AW_1 (23.7%), AW_2 (5.3%), AW_7 (6.3%) and RW_9 (2.57%), while were below 1% in the other samples. The taxa and abundance of SRB in AW samples were higher than those in RW samples. *Desulfosporosinus*, *Desulfurispora*, *Desulfitobacterium*, *Desulfovibrio*, *Desulfurispora* were detected in most of AW samples. While only *Desulfosporosinus* was detected in most of RW samples. The 11 taxa of SRB mentioned above were not detected in BW sample.

In addition, based on previous research (Wall and Krumholz, 2006; You et al., 2021), 16 taxa of microorganisms possibly related to uranium-reduction were detected for downstream analysis (Supplementary Figure S5), including *Clostridium, Arthrobacter, Pseudomonas, Rhodanobacter, Bacillus, Acidithiobacillus, Rhodopseudomonas, Cellulomonas, Desulfitobacterium, Acidovorax, Enterobacter, Thiobacillus, Geobacter, Desulfovibrio, Anaeromyxobacte*, and *Desulfotomaculum*. Compared with sulfur/sulfate-reducing bacteria, the relative abundance of uranium-reducing bacteria was generally higher. The relative abundance of most uranium-reducing bacteria in RW samples were higher than in AW samples. *Clostridium*, *Arthrobacter*, *Pseudomonas*, *Rhodanobacter*, *Bacillus* were common uranium-reducing bacteria, which were often isolated from uranium mine wastewater or groundwater and often used in bioremediation (Wall and Krumholz, 2006; You et al., 2021). In RW samples, the relative abundances of *Arthrobacter*, *Clostridium*, *Rhodanobacter* and *Pseudomonas* were 2.8, 3, 1.7, and 8 times of AW, respectively. While the relative abundance of *Bacillus* in AW groundwater was 34 times higher than that in RW.

### 3.4. Correlation analysis of microbial community and environmental factors

**Figure 8:**
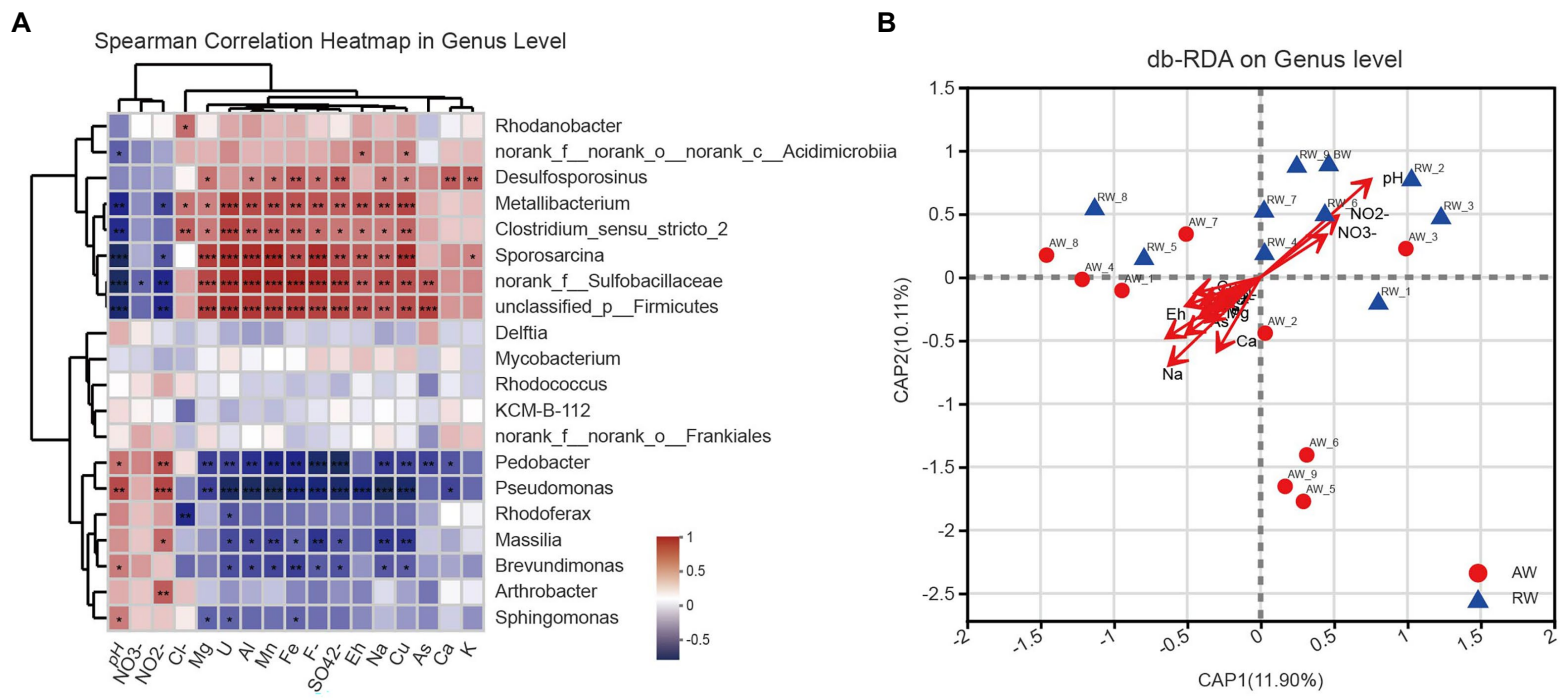
**(A)** Heatmap of spearman correlations between the relative abundance of the top 20 dominant bacterial communities and environmental factors at genus level. The *x*-axis and *y*-axis are environmental factors and taxa, respectively, and the spearman correlation *R* value and *p*-value are obtained by calculation. Red represents positive correlations between the microorganisms and the variables while Blue represents negative correlations. The *R* values are shown with different colors and the *p*-value less than 0.05 is marked with (*0.01 < *p* < 0.05, **0.001 < *p* < 0.01, ***p < 0.001). Note that NO3-, NO2-, Cl-, F-, and SO42- represent $N0_{3}^{-}$ , $N0_{2}^{-}$ , $Cl^{-}$ , $F^{-}$ , $SO_{4}^{2-}$ , respectively. **(B)** Distance-based redundancy analysis (db-RDA), derived from the Bray–urtis distance in genus level, represents the relationship between community structures and the environmental factors that influence them.

To explore the effect of environmental factor on the microbial community structures, we computed the spearman correlations between the relative abundance of the top 20 dominant bacterial communities and 17 environmental factors at genus level (Figure 8A), some important co-correlation patterns were discovered. we found that taxa could be clustered into three groups based on genus level. The first group contained eight genera, *Rhodanobacter*, *norank_f_norank_o_norank_c_Acidimicrobiia*, *Desulfosporosinus*, *Metallibacterium*, *Clostridium_sensu_stricto_2*, *Sporosarcina*, *norank_f_Sulfobacillaceae* and *unclassified_p_Firmicutes*, which tended to be positively correlated with most environmental factors, however, show strong negative correlations with pH and NO_2_^−^. The second group contained five genera that tended to be relatively weakly correlated with most environmental factors. The third group contained seven genera, *Pedobacter*, *Pseudomonas*, *Rhodoferax*, *Massilia*, *Brevundimonas*, *Arthrobacter*, and *Sphingomonas,* which tended to be negatively correlated with most environmental factors, but had strong positive correlations with pH and NO_2_^−^.

Furthermore, we used distance-based redundancy analysis (db-RDA) to assess potential impacts of the environmental factors on microbial communities. We showed the results of db-RDA between the environmental factors and microbial communities at genus level in Figure 8B. The results showed that the environmental factors with different contributions led to changes in groundwater microbial communities, among which the largest contributing environmental factor was pH and other important environmental factors were Na^+^, Eh, Ca^2+^ and NO_2_^−^, etc. Similarly, regarding the results at OTU level in Supplementary Figure S6. At OTU level, the results also showed that pH and Eh were the most influential factor affecting the composition of microbial community in the groundwater samples.

**Figure 9:**
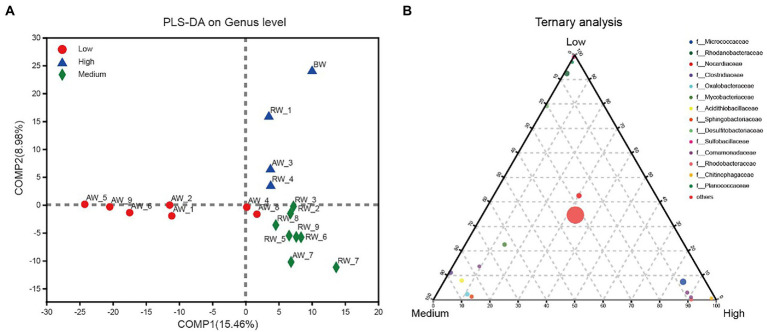
**(A)** PLS-DA analysis for three groups of groundwater samples which were divided into three groups according to the size of pH values, with pH < 3.0 (Low); 3.0 < pH < 5.0 (Medium); pH > 5.0 (High). **(B)** Ternary analysis for the groups of samples from **(A)**. In the ternary analysis, the abundances of the grouped samples were averaged, and others was the pooled set of taxa whose abundance accounted for less than 0.03%.

We further investigated the effect of pH distribution on microbial community structures. According to the size of pH values, we firstly divided the groundwater samples into three groups with pH < 3.0 (Low), 3.0 < pH < 5.0 (Medium), pH > 5.0 (High). The results of PLS-DA analysis showed that these groundwater samples could be clearly distinguished and clustered into three groups, indicating that the composition of bacteria at genus level under different pH gradients was different (Figure 9A). Furthermore, we revealed the microbial composition characteristics at genus level under different pH gradients by using ternary analysis (Figure 9B). The results showed that the composition and distribution ratio of microorganisms in different groundwater samples were different. It was mainly from *Sulfobacillaceae*, *Rhodanobacteraceae*, Planococcaceae, *Desulfitobacteriaceae* in the low pH group; *Clostridiaceae*, *Oxalobacteraceae*, *Acidithiobacillaceae*, *Sphingobacteriaceae* and Mycobacteriaceae in the middle gradient pH group; *Micrococcaceae*, *Comamonadaceae*, *Rhodobacteraceae*, and *Chitinophagaceae* in these samples with high pH values.

## 4. Discussion

In the study, the distribution of environmental factors in AW samples is very different from each other, especially SO_4_^2−^, U, Fe, Mn, Al^3+^, and other heavy metals, while the distribution of environmental factors in RW samples is similar (Figure 2). Additionally, we found that the diversity of AW samples was generally higher than that of the RW samples (Figures 3C, 6), indicating that the differences in diversity may reflect more microbial niches in the anaerobic subsurface. The pH value of the groundwater in ore-bearing beds ranged from 2.15 to 5.86, although most values were between 2 and 3. Such a wide pH range of the groundwater in ore-bearing beds led to relatively large differences in physicochemical parameters in AW samples, including metal ions such as U, Fe, Mn and anions such as SO_4_^2−^, F^−^, especially the Eh values. These environmental factors play an important role in shaping the microbial community. Carlson et al.’s (2019) study of a contaminated aquifer in Oak Ridge, Tennessee, United States, found that microbial communities of the *Rhodanobacter* genus were abundant in the most contaminated well, and they also found that low pH and high metal-contaminated are likely selective pressures on the microbial community. Yan’s research also shows that environmental selection is a key mechanism driving the spatial differentiation of groundwater microbial communities (Yan et al., 2020). In our research, the selection pressure of environmental factors in ore bearing groundwater probably drive more microbial niches.

The microbial community composition in AW and RW samples was different (Figures 5, 7A). Ore-bearing groundwater usually come from more oligotrophic and oxygen-poor environments. Most of the electron donors for biological growth derived from hydrogen sulfide, methane or reduced iron, and electron acceptors are mainly from sulfate. Thus, these conditions contributed to the high abundance of reducing and autotrophs microorganisms in the ore-bearing layers, such as *Desulfosporosinus* and *Sulfobacillus* (Figure 5B). It was reported that *Desulfosporosinus* often isolated from acid mine wastewater or pits, which has acid resistance, metal resistance and sulfate-reducing (Sánchez-Andrea et al., 2015; Mancini et al., 2016; Panova et al., 2021). Sulfobacillus is considered sulfur oxidizer and usually applied to mineral bioleaching (Ni et al., 2014). In addition, the low pH of AW determined the growth of some acidophilic, such as Acidibacillus, Acidithrix. Bacillus have higher proportion in AW samples indicating that it has good adaptability in extreme acidic environment. The study shown that Bacillus can remain active for more than 2 years in an extremely acidic environment (pH = 1.5; Chen et al., 2019). In the other side, the residual water in the pipe easily dissolves oxygen from the atmosphere and obtains organic matter from the inflow of external rainwater, Organic matter can be used as an electron donor for microbes. Thus, compared with the AW samples, the RW samples contained more heterotrophic microorganisms, such as *Pedobacter*, *Pseudomonas*, *Massilia*, *Brevundimonas*, and *Sphingomonas* (Figure 5B). Among them, *Pseudomonas* and *Pedobacter* have been reported to have the ability to remove metal pollution (Wall and Krumholz, 2006; Field et al., 2018; You et al., 2021). Therefore, in addition to dilution by external water sources and physical and chemical precipitation, microorganisms may play a role in the reduction of metal pollution in RW samples.

At OTU level, the most environmental factors affecting the microbial community was Eh in AW samples (Supplementary Figure S6), indicating that variations in Eh will strongly select for organisms with anaerobic respiratory metabolisms. This is consistent with previous findings that microbial community composition is more affected by redox potential in a historical uranium-mining site (Sitte et al., 2015). Under anaerobic conditions, microorganisms will preferentially utilize electron acceptor with higher Eh (such as nitrate) in the environment for anaerobic respiration, while the reduction process of electron acceptor with lower Eh will be inhibited (North et al., 2004). On the other side, pH is more important to influencing the microbial community in the RW samples because apart from variations in pH, there are few other changes in geochemistry. Furthermore, our study shows that the composition of the microbial community is clearly pH-dependent (Figure 9) and has correlation with other environmental factors (Figure 8). Indicating that pH value and other environmental factors can strongly affect the distribution of microorganisms and form different ecological niches. In our research, the *Desulfosporosinus* has the highest proportion in the most sulfate-contaminated well (23%), and was positively correlated with sulfate concentration. It was reported that the sulfite reductase gene (*dsrAB*) was most prevalent in wells contaminated with high concentrations of sulfate (Waldron et al., 2009). Under low pH and high sulfate concentration, sulfate-reducing bacteria may use sulfate as electron acceptor for their own growth, Therefore, sulfate-reducing bacteria may be better able to survive in such conditions than other microbial genera.

Above all, in the AW samples, the discovery of high abundance of *Desulfosporosinus* and *Sulfobacillus* indicating that the environmental of the ore-bearing layer is changing from an oxidizing environment during mining to a more reducing environment suitable for the growth of reducing microorganisms. This has important implications for the remediation of groundwater in the study area. In addition, compared with the Eh value of the adjacent active acid leaching area (437.6 ~ 442.2 mv), the Eh value in this study area (93.7 ~ 373.1mv) is lower, which further proves that the decommissioned mining area is changing to a more reductive state. However, as a whole, it is uncertain whether the whole system will continue to focus on reduction (promoting uranium immobilization) or oxidation (promoting uranium solubilization). In our study, the ore bearing aquifer was still dominated by extremely acidic environment and contains high concentration of SO_4_^2−^, which may promote dissolve insoluble U (IV) from ores. With the increase of the decommissioning time, if the groundwater in the decommissioning mining area is continuously affected by the groundwater not exploited in the upstream, the pH value of the groundwater in the decommissioning mining area will gradually rise and eventually tend to the background value. From this point of view, the whole decommissioning groundwater system will continue to change from an oxidizing state to a more reductive state. However, in the process of the whole system changing from oxidizing state to reducing state, if the upstream groundwater carries oxidants to supplement the groundwater system in the decommissioned mining area, the oxidation degree of the system will be temporarily improved, thus affecting the migration of variable valence metals such as uranium in the aquifer.

## 5. Conclusion

In the study, we have reported the occurrence characteristics of microbial communities in the groundwater of a decommissioned acid *in-situ* uranium mine and their connection to environmental factors. Through the research, we draw the following conclusions:

1. Differences in groundwater environmental factors (including pH, sulfate, and heavy metals) in ore-bearing aquifer leading to more microbial diversity, while the water quality of pipe well groundwater was similar, resulting in a similar microbial community composition.
2. pH is more important to influencing the microbial community in the RW samples because apart from variations in pH, there are few other changes in geochemistry. In contrast, in the AW samples, there are variations in Eh, which will strongly select for organisms with anaerobic respiratory metabolisms.
3. There are relatively high abundances of *Sulfurobacillus* and *Desulfosporosus* in the groundwater of the ore-bearing aquifer (AW), indicating that the groundwater environment has changed from an oxidized state in the past to a more reduced state in the direction of recovery.

These results extend our understanding of groundwater microbial community changes in the decommissioned acidic in-situ uranium mine and have potentially positive implications for the search for effective bioremediation methods for groundwater.

## Data availability statement

The datasets generated for this study can be found in the NCBI Sequence Read Archive, accession number PRJNA901217.

## Author contributions

FZ: conceptualization, data curation, investigation, software, and writing—original draft. BZ: data curation, investigation, and writing—review and editing. WM: funding acquisition, writing—review and editing. JL: conceptualization, funding acquisition, and supervision. All authors contributed to the article and approved the submitted version.

## Funding

This work was supported by the National Nuclear Facility Decommissioning and Radioactive Waste Disposal project (No. 1276), and the National Natural Science Foundation of China (Nos. 62262069 and 61802157), and the Open Project Program of Yunnan Key Laboratory of Intelligent Systems and Computing (No. ISC22Z03).

## Conflict of interest

The authors declare that the research was conducted in the absence of any commercial or financial relationships that could be construed as a potential conflict of interest.

## Publisher’s note

All claims expressed in this article are solely those of the authors and do not necessarily represent those of their affiliated organizations, or those of the publisher, the editors and the reviewers. Any product that may be evaluated in this article, or claim that may be made by its manufacturer, is not guaranteed or endorsed by the publisher.

## Footnotes

^1^www.majorbio.com

^2^https://www.r-project.org/
