# Supplementary material for: Characteristics of groundwater microbial communities and the correlation with the environmental factors in a decommissioned acid in-situ uranium mine
Source: Front Microbiol. 2023 Feb 22;13:1078393. doi: 10.3389/fmicb.2022.1078393 (PMC9992980; doi:10.3389/fmicb.2022.1078393)
Supplement: Supplementary file 2 [file Data_Sheet_1.PDF]

Supplementary Materials for

Characteristics of Groundwater Microbial Communities and the Correlation with the Environmental Factors in a Decommissioned Acid In-Situ Uranium Mine

Fangfang Zhu[1#], Bei Zhao[2#], Wenwen Min[3*], Jiang Li[*1],

[1]State Key Laboratory of Nuclear Resources and Environment, East China University of Technology, Nanchang, Jiangxi, 330013, China

[2]China University of Geosciences (Beijing), Beijing 100083, China

[3]School of Information Science and Engineering, Yunnan University, Kunming, Yunnan, 650091, China.

*** Correspondence:**

Wenwen Min, Jiang Li

minwenwen@ynu.edu.cn; li66001@163.com

Contents

S1. Statistical analysis on 16S rRNA gene sequencing data……………………2

S2. Supplementary figures……………………………………………………3

S3. Supplementary tables………………………………………………………9

S1. Statistical analysis on 16S rRNA gene sequencing data

In this study, A total of 876,084 valid sequence reads were collected from the 19 groundwater samples after quality filtering. These sequence reads were classified into 3,031 valid OTUs with a similarity of 97%. Taxa annotation results statistics: Domain: 1, Kingdom: 1, Phylum: 46, Class: 128, Order: 288, Family: 458, Genus: 898, Species: 1565, OTU: 3031, and the corresponding microbial count data were presented in Tables S1 to S5. The total OTUs of AW, RW, BW samples were 2,247, 1,440 and 432, respectively.

S2. Supplementary figures

In this paper, there are 6 supplementary pictures.

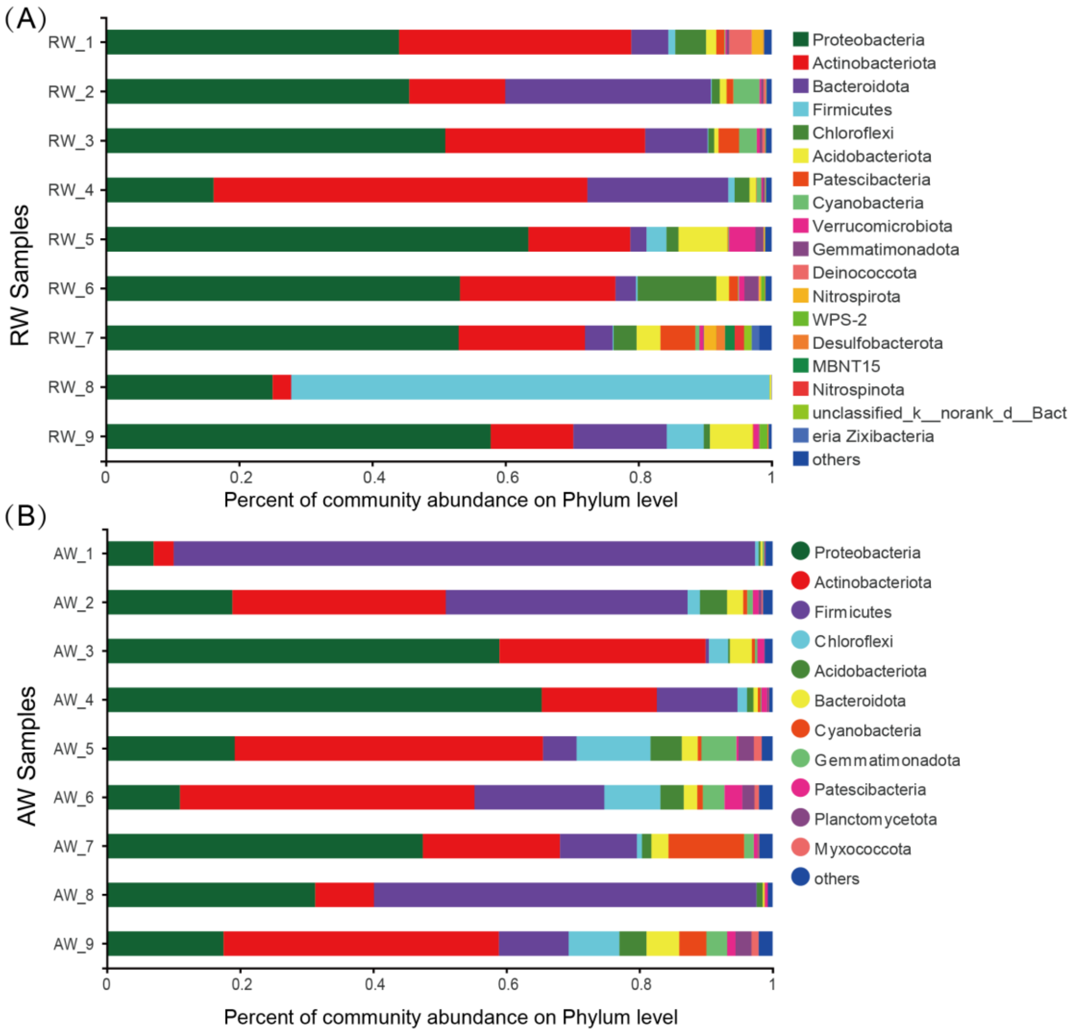

Figure S1. (A) A total of 37 phyla were detected in RW samples, of which the most abundant 10 phyla accounted for 98%; mainly Proteobacteria (45.4%), *Actinomycetes* (23.2%), *Bacteroidetes* (10.1%); (B) A total of 43 phyla were detected in AW samples, the most abundant 10 phyla accounted for 98.2% of the total microbial community; Mainly *Proteobacteria* (30.7%), *Actinomycetes* (27.2%) and *Firmicutes* (26.7%); And *Firmicutes* accounted for the largest proportion in AW_1 and AW_8 samples.

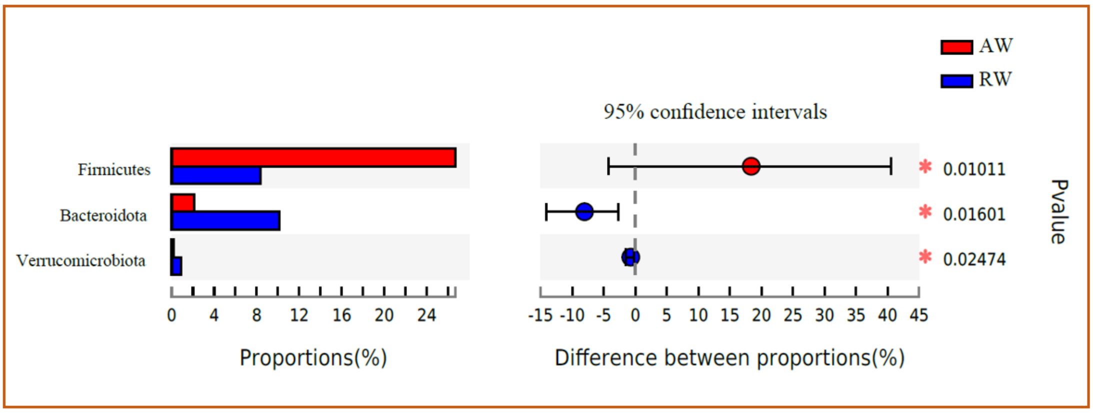

Wilcoxon rank-sum test bar plot on Phylum level

Figure S2. Wilcoxon rank-sum test bar plot on phylum level. *Firmicutes* and *Bacteroidota* had significant differences in AW and RW samples. Compared with other taxa, *Firmicutes* only had higher relative abundance in AW_1 (87.5%), AW_2 (36.2%), AW_8 (57.5%), RW_8 (71.8%) samples.

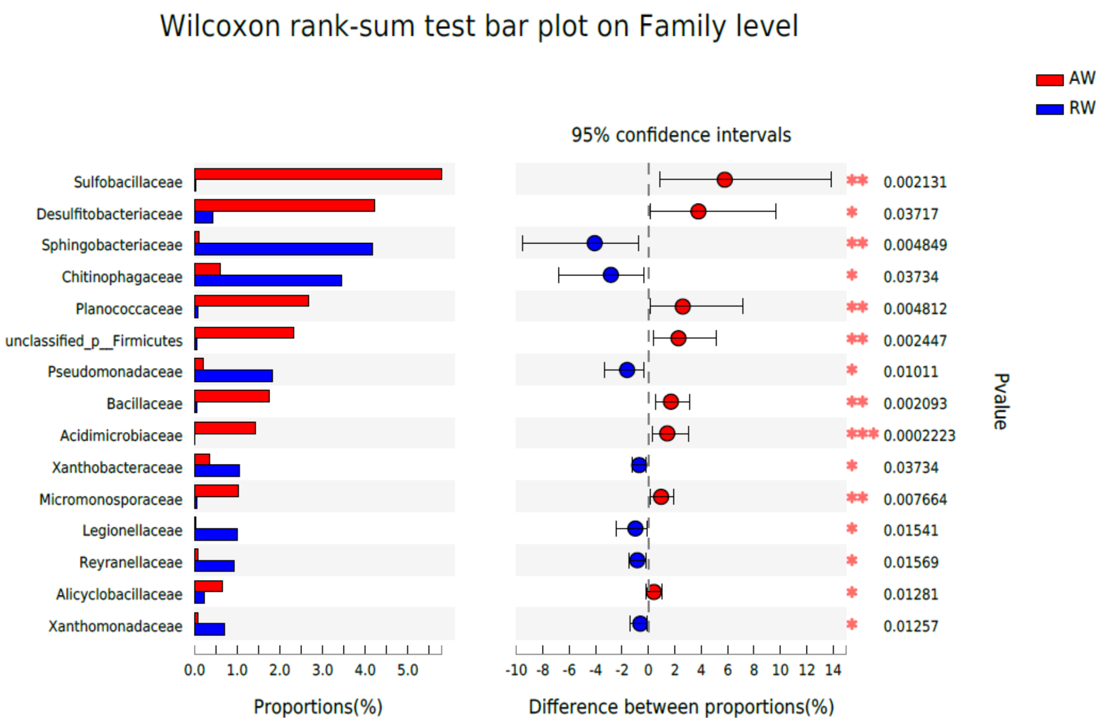

Figure S3. Differential abundance analysis. Wilcoxon rank-sum test bar plot on family level. Only differentially abundant genus is shown. The y-axis represents the taxa name, the x-axis represents the average relative abundance of taxa in different groups, and the far right is the P value, * 0.01≤P≤0.05, ** 0.001≤P≤0.01, ***P ≤ 0.001.

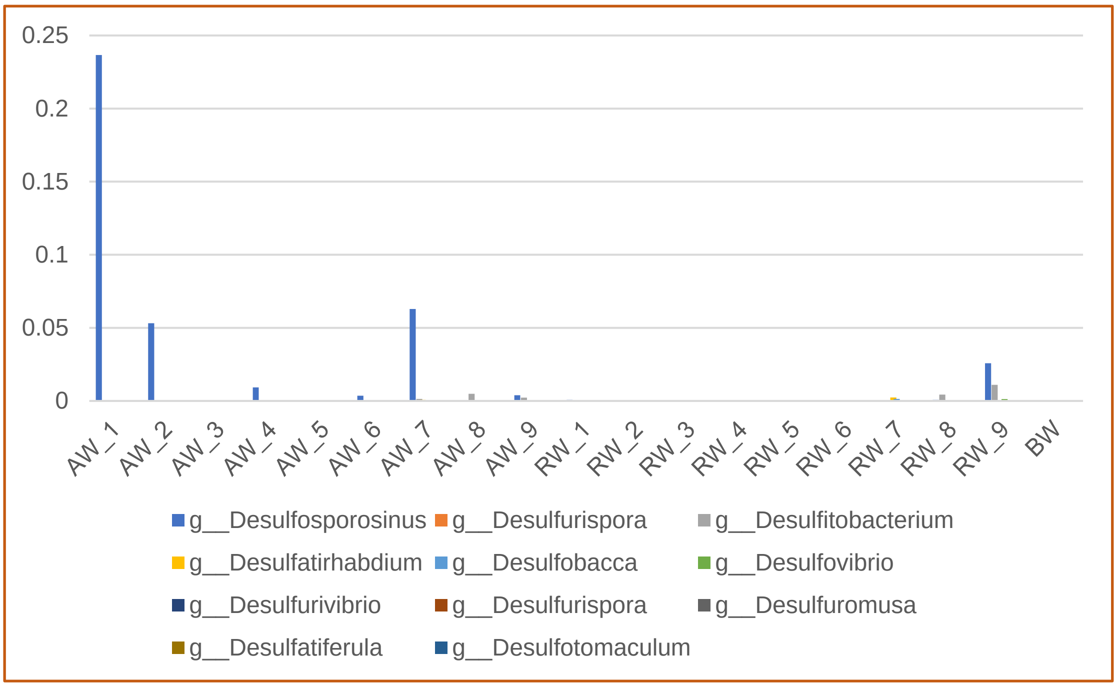

Figure S4. The relative abundance of most sulfate-reducing bacteria genera in each sample was low, *Desulfosporosinus* was detected in all samples of AW and RW, but only with relatively high abundance in AW_1 (23.7%), AW_2 (5.3%), AW_7 (6.3%), RW_9 (2.6%). The taxa and abundance of sulfate-reducing bacteria in the AW samples were obviously higher than those in the RW samples. Except for *Desulfatiferula*, the rest of the sulfate-reducing bacteria were detected in the AW samples. *Desulfuromusa* and *Desulfotomaculum* were not detected in RW samples, and the rest were only distributed in a few samples. The 11 taxa of sulfate-reducing bacteria mentioned above were not detected in the BW sample.

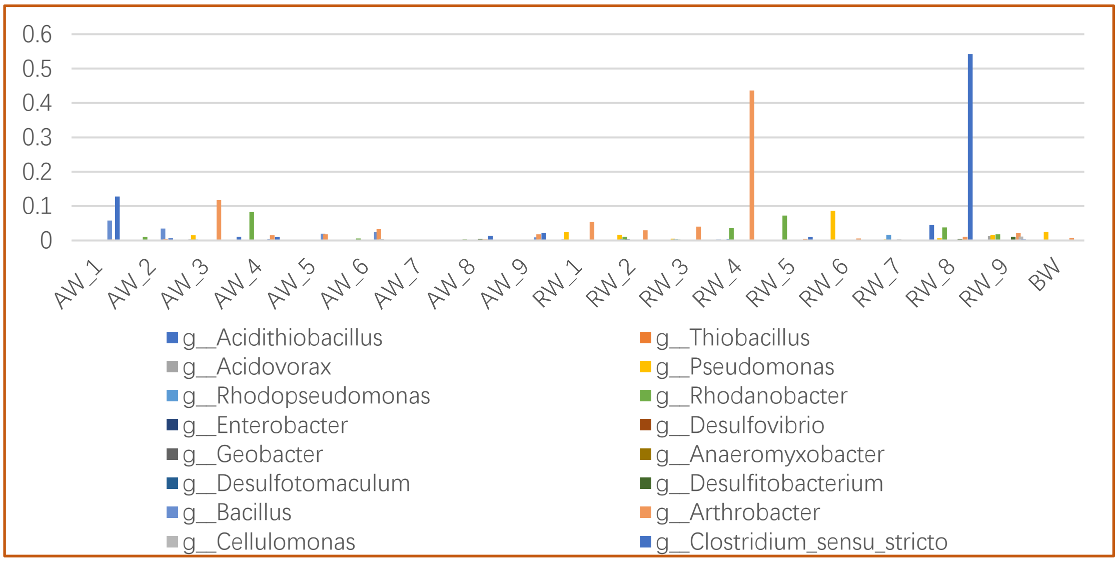

Figure S5. Compared with sulfate-reducing bacteria, the relative abundance of uranium-reducing bacteria was generally higher than the former, And the variety in the BW sample was also relatively rich. The distribution of most uranium-reducing bacteria was not significantly different between the AW and RW samples, and only a few taxa were obviously different in distribution. The abundance of *Clostridium* was higher in AW_1 (12.8%) and RW_8 (54.2%) samples, respectively; The relative abundance of *Arthrobacter* was higher in AW_3 (11.7%) and RW_4 (43.6%) samples; *Pseudomonas* were more abundant in RW_6 (8.6%) samples *Rhodanobacter* had relatively high abundance in AW_4 (8.25%) and RW_5 (7.25%) samples, respectively.

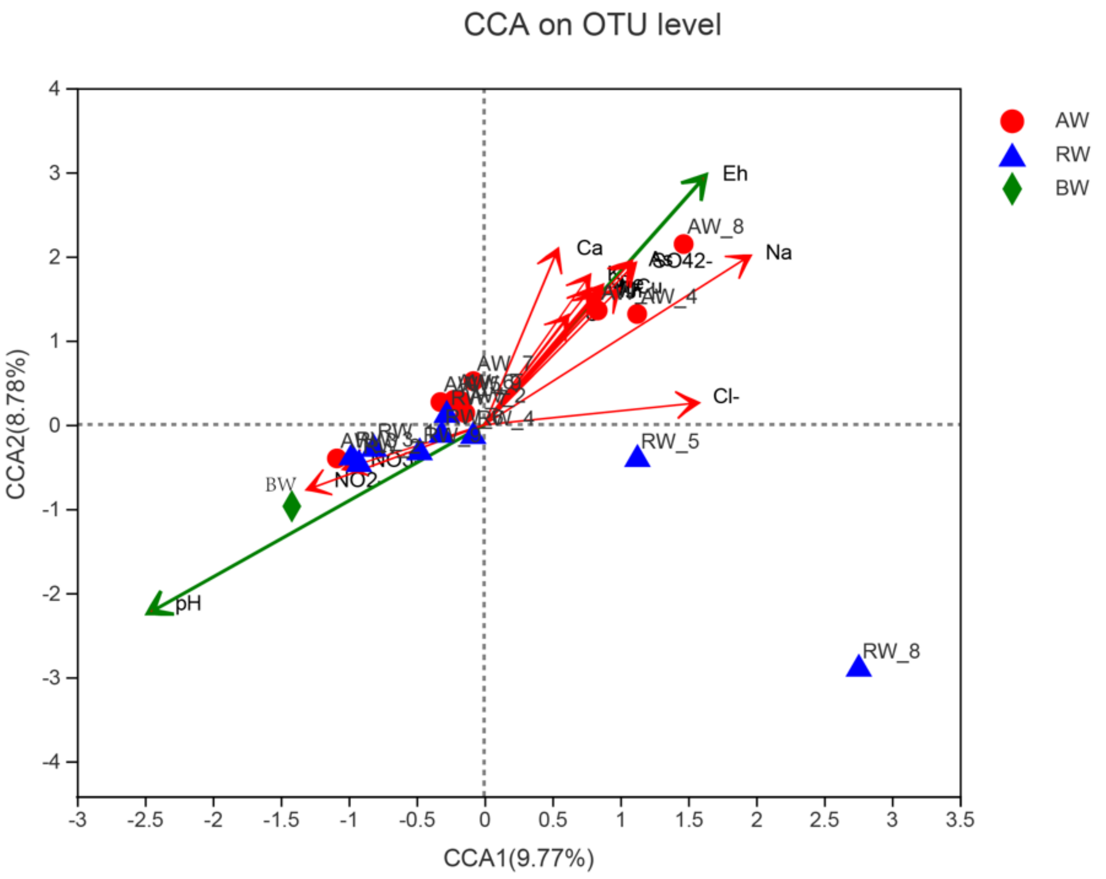

Figure S6. Canonical correspondence analysis (CCA) is mainly used to reflect the relationship between microorganisms and environmental factors. The above figure is the correlation analysis of microorganisms and environmental factors based on the OTU level. The results show that pH value was the most influential factor affecting microbial composition, followed by Eh value, and mainly affected the microbial community distribution in RW and AW samples, respectively.

S3. Supplementary tables

In this paper, there are a total of 8 supplementary tables and all tables were presented in an Excel file (See Supplementary Tables.xlsx).

We collected 19 groundwater samples and the corresponding microbial count data based on the high throughput 16S rRNA gene sequencing method were presented in Tables S1 to S6.

Table S1. otu_taxon_otu.

Table S2. otu_taxon_phylum.

Table S3. otu_taxon_Class.

Table S4. otu_taxon_order.

Table S5. otu_taxon_family.

Table S6. otu_taxon_genus.

Table S7. Values of the groundwater environmental factors in the RW, AW and BW samples.

Table S8. Alpha-diversity indices for the groundwater samples.

Table S9. Spearman correlation coefficient of the microbial diversity indices and physicochemical factors in groundwater samples, where '*' indicates significant differences at $P<0.05$.
